# Generation of an mESC model with a human hemophilia B nonsense mutation via CRISPR/Cas9 technology

**DOI:** 10.1186/s13287-022-03036-2

**Published:** 2022-07-26

**Authors:** Yanchun Ma, Wenwen Sun, Lidong Zhao, Mingze Yao, Changxin Wu, Pengfei Su, Linhua Yang, Gang Wang

**Affiliations:** 1 Department of Hematology, The Second Hospital of Shanxi Medical University, Taiyuan, 030001 Shanxi Province China; 2 Institutes of Biomedical Sciences, Shanxi University, Taiyuan, 030006 Shanxi Province China

**Keywords:** Hemophilia, Mouse embryonic stem cell, Nonsense mutation, CRISPR/Cas9 technology, Hepatocyte-like cell

## Abstract

**Background:**

Hemophilia B is a rare inherited genetic bleeding disorder caused by a deficiency or lack of coagulation factor IX, the gene for which (*F9*) is located on the X chromosome. Hemophilia B is currently incurable and the standard treatment is coagulation factor replacement therapy. Although gene therapy has the potential to cure hemophilia, significant barriers are still needed to be overcome, e.g., off-target effects and immunoreactivity, so new approaches must be explored. Nonsense mutations account for 8% of all the hemophilia B mutation types and can result in the development of coagulation factor inhibitors. In this study, CRISPR/Cas9 technology was used to construct a mouse embryonic stem cell model with a hemophilia B nonsense mutation (*F9 c.223C* > *T*) in humans to investigate the pathogenesis and treatment of nonsense mutations in hemophilia B.

**Methods:**

First, a donor plasmid with a mutation (*F9 c.223 C* > *T*) and sgRNAs were constructed. Second, both the donor plasmid and the px330-sgRNA were electroporated into mouse embryonic stem cell, and the mutant cells were then screened using puromycin and red fluorescence. Third, the mutant cell lines were tested for pluripotency and the ability to differentiate into three layers. Finally, the effect of mutation on gene function was studied in the differentiation system.

**Results:**

The mutant vector and effective sgRNA were constructed, and the mutant cell line was screened. This mutant cell line exhibited pluripotency and the ability to differentiate into three layers. This point mutation affects F9 expression at both the RNA and protein levels in the differentiation system.

**Conclusions:**

The mutant cell line obtained in the current study had a single-base mutation rather than a base deletion or insertion in the exon, which is more similar to clinical cases. In addition, the mutant has the characteristics of mouse embryonic stem cells, and this point mutation affects *F9* gene transcription and translation, which can be used as a disease model for studying the pathogenesis and treatment of hemophilia at the stem cell level.

**Supplementary Information:**

The online version contains supplementary material available at 10.1186/s13287-022-03036-2.

## Background

The clustered regularly interspaced short palindromic repeats (CRISPR)/Cas9 gene-editing system has become a powerful genome-editing tool in the field of molecular biology since its discovery. When introduced into cells, the Cas9 protease is directed to the target genome sequence by a specific synthetic sgRNA and subsequently breaks both DNA strands. The resulting double-strand breaks are then repaired by either the error-prone nonhomologous end-joining or homology-directed repair, resulting in genomic DNA knock-out or knock-in [1, 2].

Hemophilia B is a rare X-linked bleeding disorder caused by a deficiency or lack of coagulation factor IX (FIX). The *F9* gene is located on the X chromosome. Hemophilia B is recessively inherited and is predominantly expressed in males because males have only one X chromosome [3, 4]. The type of causative mutation determines the severity degree of hemophilia, which can be classified into three levels based on the residual FVIII or FIX activity as follows: severe (< 1 international unit [IU]/dl), moderate (1–5 IU/dl), or mild (6 to < 40 IU/dl) [5, 6]. The standard therapy for severe patients in developed countries requires prophylactic treatment with replacement factors, while patients with severe conditions in developing countries are treated mainly on demand [7]. The most serious complication of hemophilia B replacement therapy is the development of neutralizing antibodies (inhibitors) against FIX. At high titers, these inhibitors may reduce the efficacy of replacement factors or even render replacement therapy completely ineffective, increasing the burden on family and society [8]. Although gene therapy has the potential to cure this disease, factors (e.g., immunogenicity and off-target effects) remain unresolved.

Nonsense mutations may increase the risk for the development of coagulation factor inhibitors [9]. The hemophilia B mutation database (http://www.factorix.org/) revealed 87 different forms of nonsense mutations, accounting for 8% of all hemophilia B mutation types. An extensive meta-analysis study based on the Human Gene Mutation Database showed that 11% of all genetic disease-causing mutations are nonsense mutations resulting in the translation of premature termination codons (PTCs) [10]. Therefore, a new therapeutic strategy for hemophilia with a nonsense mutation should be developed.

Truncated proteins are produced when PTCs are introduced into nonsense mutations, resulting in gene inactivation. Nonsense mutations may also reduce messenger RNA levels due to the rapid mRNA turnover caused by the nonsense-mediated mRNA decay pathway [11]. Aminoglycosides and several small molecular compounds can bind to the ribosome decoding center, reducing the accuracy of codon and anticodon pairing, promoting PTC binding to near-cognate aminoacyl tRNA, and decoding PTC into a meaningful codon. Protein synthesis produces a constant number of functional full-length proteins, which is used in translational bypass therapy for nonsense mutations [12, 13].

The effect of read-through therapy is related to the nonsense mutant gene mRNA stability. A strong read-through effect can be acquired only when the mRNA (PTC-mRNA)-containing PTC is stable [14, 15]. New compounds that can promote the premature stop codon read-through in nonsense mutations have been recently developed. For instance, ELX-02, which is designed to treat genetic diseases caused by nonsense mutations (e.g., cystic fibrosis and cystinosis) is currently being studied in clinical trials. These synthetic small molecular drugs have shed light on the treatment of hemophilia B. To investigate read-through treatment, the present study aimed to generate an *F9* nonsense mutation mouse embryonic stem cell (mESC) that is closer to clinical cases without gene deletion or insertion but only a single-base mutation. This cell model is also useful for studying the mechanism and treatment of hemophilia B.

Since a nonsense mutation of *F9 c.223C* > *T* (*p.R75X*) was detected in a patient with hemophilia B at the hospital of the current study and the mouse with this mutation encodes the same amino acid as humans, an mESC with this mutation was constructed.

## Methods

### Plasmid construction

The primers were designed to amplify and purify the left and right arms, as well as the Loxp-PGK-puro-p2A-Mcherry-Loxp via polymerase chain reaction (PCR), with the mutation point on the left arm (F9 c.223C > T). The donor plasmids (PGGA-M. LA-LOXP-PGK-Puro-P2A-Mcherry-LOXP-RA) were assembled using the Golden Gate method (E1601 NEB, BsaI-HFV_2_). The sgRNA was designed on the website [16] (https://zlab.bio/guide-design-resources) and inserted into the pX330 plasmid, which targets the second intron of the F9 gene. The vector sequences are shown in Additional file 2: Text 1, and the primers’ sequences are listed in Table 1.

**Table 1:** Primer sequence information for vector construction and RT-PCR

	Target	Forward/reverse primer (5′–3′)
House-keeping genes (RT-qPCR)	*Gapdh*	AACTTTGGCATTGTGGAAGGGCTCA/ TTGGCAGCACCAGTGGATGCAGGGA
Pluripotency marker (RT-qPCR)	*Nanog*	CTCAAGTCCTGAGGCTGACA/ TGAAACCTGTCCTTGAGTGC
Pluripotency marker (RT-qPCR)	*Oct4*	TAGGTGAGCCGTCTTTCCAC/ GCTTAGCCAGGTTCGAGGAT
Pluripotency marker (RT-qPCR)	*Sox2*	CTGCAGTACAACTCCATGACCAG/ GGACTTGACCACAGAGCCCAT
Pluripotency marker (RT-qPCR)	*Klf4*	AACATGCCCGGACTTACAAA/ TTCAAGGGAATCCTGGTCTTC
Endoderm marker (RT-qPCR)	*Sox17*	CGAGCCAAAGCGGAGTCTC/ TGCCAAGGTCAACGCCTTC
Endoderm marker (RT-qPCR)	*Gata4*	CCCTACCCAGCCTACATGG/ ACATATCGAGATTGGGGTGTCT
Mesoderm marker (RT-qPCR)	*T*	CATCGGAACAGCTCTCCAACCTAT/ GTGGGCTGGCGTTATGACTCA
Mesoderm marker (RT-qPCR)	*MixL1*	ACGCAGTGCTTTCCAAACC/ CCCGCAAGTGGATGTCTGG
Ectoderm marker (RT-qPCR)	*Pax6*	GCAGATGCAAAAGTCCAGGTG/ CAGGTTGCGAAGAACTCTGTTT
Ectoderm marker (RT-qPCR)	*Nestin*	CCCTGAAGTCGAGGAGCTG/ CTGCTGCACCTCTAAGCGA
Define endoderm marker (RT-qPCR)	*Hnf-4α*	GCCAACGATCACCAAGCAAG/ GCCAAAAGGCTCACACCAAAG
Hepatoblast marker (RT-qPCR)	*Afp*	CGTGATGCTTTGGGCGTTTA/ GCCAAAAGGCTCACACCAAAG
Hepatocyte-like cell marker (RT-qPCR)	*Ttr*	CCCTGCTCAGCCCATACTCCTA/ TGCTTTGGCAAGATCCTGGT
Hepatocyte-like cell marker (RT-qPCR)	*Alb*	AAACCTTGTCACTAGATGCAAAGACG/ GGGTAGCCTGAGAAGGTTGTGG
Target gene (RT-qPCR)	*F9*	TGGAAGCAGTATGTTGATGGAGA/ CCAACTTGGCACCAGCATTC
Guide RNA (px330)	*Intron 2*	CACCGAAAGGAGTGTGGAACTAGAG/ AAACCTCTAGTTCCACACTCCTTTC
Blunt-sequencing (PCR)	Left arm (1176 bp)	CATGAATTTCAGTGAAATGCTTCCATCACTGGC/ CCTGTGTATGTACTACTCAAAAAGGTCAGAAGAGAGTG
Blunt-sequencing (PCR)	Blunt-Left arm- Mutation (3032)	GAAGAAAGATGTAGTTTTGAAGAAGCATGAGAAG/ CTTCTCATGCTTCTTCAAAACTACATCTTTCTTC
Blunt-sequencing (PCR)	PGK-puro (1145 bp)	CCCAAGTAATAGGGGTAGGGGAGGCGCTTTTCCCAAGGCAGTCTG/ GAAGTTAGTGGCGGCACCGG GCTTGCGGGTCATG
Blunt-sequencing (PCR)	P2A-mcherry (988 bp)	CAAGCCCGGTGCCGCCACTAACTTCAGCTTGTTGAAGCAG/ GGTCAGGGTGCAGGCAGTGAAAAAAATGCTTTATTTGTGAAATTTGTG
Donor construction (PCR)	PGGA-Left arm(842 bp)	GGCTACGGTCTCTGGAGCAAACAGGCTTCTGTCCTTC/ GGCTACGGTCTCATACGAAGTTATCCTGTGTATGTACT ACTCAAAAAG
Donor construction (PCR)	Loxp-PGK-puro (1161 bp)	GGCTACGGTCTCACGTATAATGTATGCTATACGAAGTTATGGGTAGGG/ GGCTACGGTCTCCTTTCCAGGAAGGCGGGCACCCC
Donor construction (PCR)	*mCherry-loxp* *(943 bp)*	GGCTACGGTCTCCGAAACCTCCGCGCCCCGCAAC/ GGCTACGGTCTCGAACTTCGTATAGCATACATTATACG AAGTTATTTACT
Donor construction (PCR)	PGGA-Right arm(758 bp)	GGCTACGGTCTCGAGTTATGTGCATGTATGCACACAGATAGAA/ GGCTACGGTCTCGATGGGACCAGAACACATGTGTCTAAAATGACAG
F9-Flank	*F9*	CATGAATTTCAGTGAAATGCTTCCATCACTGGC/ TGCTACTTCCATTTGTCACGTCCTGC

### Cell culture

The E14 mESCs were cultured on gelatin-coated plates in Dulbecco’s Modified Eagle’s Medium–high glucose (DMEM, HyClone, Logan, UT, USA) supplemented with 20% fetal bovine serum (FBS, Gibco, ThermoFisher Scientific, Waltham, MA, USA), nonessential amino acids (1 mM, Gibco), GlutaMAX (1×, Gibco), sodium pyruvate (1 mM, Gibco), penicillin (50 units/mL), streptomycin (50 μg/mL, Hyclone), β-mercaptoethanol (0.1 mM), leukemia inhibitory factor (1000 U/mL), and 2i inhibitors (3 μM CHIR99021 and 1 μM PD0325901). The plates were incubated at 37 °C in a humidified incubator with 5% CO_2_, and the media was changed daily. The cells were detached using a 0.25% trypsin–EDTA solution (Gibco) at a 1:10 split ratio when the confluence reached 70%.

### Generation of F9 mutation in mESCs

**Fig. 1:**
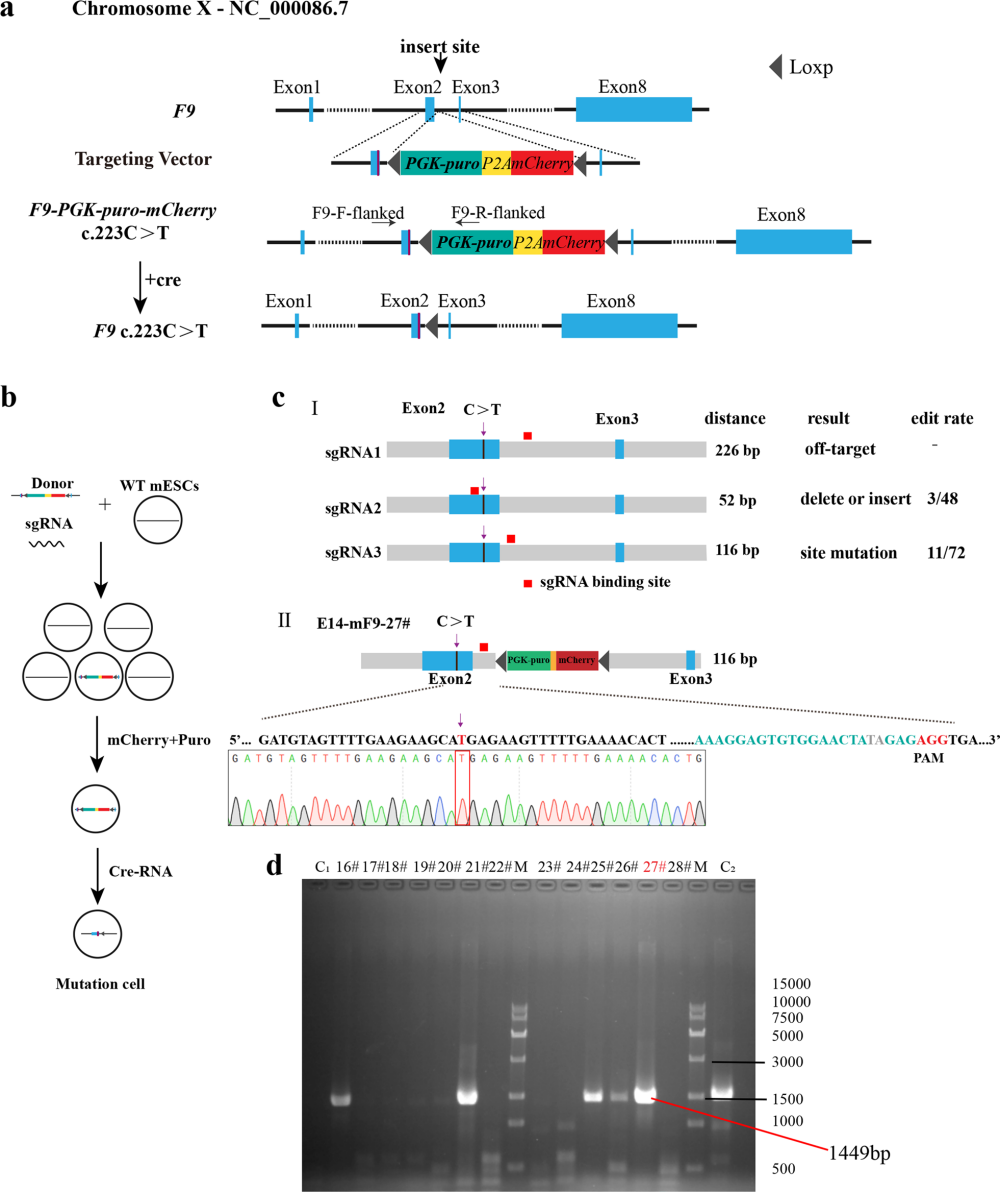
Gene mutation using CRISPR/Cas9 technology and screening of mutant cells. **a** Gene pattern diagram. **b** The experimental procedure. **c** (**I**) Editing rates of the three sgRNA, the binding position of each sgRNA is different. The numbers represent the distance from the downstream of the mutation site to that of sgRNA, and **c** (**II**) after sgRNA3 and donor transfection, the mutant cell line was screened out (E14-mF9-27#). **d** After donor and sgRNA3 transfection, partial PCR results of flanking primer (PCR product) was 1449 bp. 27# (E14-mF9-27#), M DNA marker, C1 represents negative control, and C2 represents near-positive control

Using the Ltd.P3 Primary Cell 4D-NucleofectorTM X Kit L (V4XP-3012, Lonza Group AG, Basel, Switzerland), 1 × 10^6^ mESCs were electroporated with a mixture of 6 and 2 μg donor and sgRNA-pX330 plasmids, respectively. After electroporation, the cells were cultured in a 10-cm culture dish for 12 h and screened with 1 μg/mL puromycin (Gibco) for two passages, refreshing the media every day. When the confluence reached 70%, individual clones with higher fluorescence were selected for further screening on 48-well gelatin-coated plates (one clone per well). The genomic DNA was extracted using the TIANamp genomic DNA kit (Tiangen Biotech Co., Ltd., Beijing, China). The individual clones were further verified using “*F9*-F-flanked” and “*F9*-R-flanked” primers (Fig. 1a), which confirmed that the modification in *F9* intron 2 was caused by CRISPR/Cas9-mediated donor insertion.

### Immunocytochemistry

The cells were washed twice with phosphate-buffered saline (PBS) and fixed in paraformaldehyde (4%) for 15 min at room temperature. The cells were then washed twice with PBS, permeabilized in PBS containing 0.3% Triton X-100 for 10 min, washed thrice with PBS, then blocked in 5% bovine serum albumin (BSA) at 37 °C for 1 h. The cells were then incubated overnight at 4 °C with rabbit anti-NANOG and mouse anti-OCT4 diluted in 1% BSA. The cells were then washed thrice with PBS for 5 min each to remove any unbound primary antibodies and incubated for 1.5 h with fluorescein-conjugated antirabbit and antimouse secondary antibodies (both 1:400) in 1% BSA. The cells were then washed thrice with PBS, stained with 4′,6-diamidino-2-phenylindole (1 μg/mL for 10 min), washed three more times with PBS, and imaged using a confocal laser scanning microscope (Zeiss LSM 710). The antibodies used in the present study are listed in Table 2.

**Table 2:** Reagents details

	Antibody	Dilution	Company Cat # and RRID
*Antibodies used for immunocytochemistry*			
Primary antibodies	Rabbit anti-NANOG	1:500	Novus Biologicals Cat #NB100-58842 RRID: AB_877697
Primary antibodies	Mouse anti-OCT3/4	1:200	Santa Cruz Biotechnology Cat#sc-5279 RRID: AB_628051
Primary antibodies	Rabbit anti-Factor IX	1:500	Invitrogen Cat# PA5-22304 RRID: AB_11153706
Secondary antibodies	Goat anti- Mouse IgG (H + L) Alexa Fluor 488	1:500	Thermo Fisher Scientific Cat# A-11001 RRID: AB_2534069
Secondary antibodies	Goat anti- Rabbit IgG (H + L) Alexa Fluor 488	1:500	Thermo Fisher Scientific Cat# A-11008 RRID: AB_143165
*Antibodies used for Western blot*			
Primary antibodies	Rabbit anti-Factor-IX	1:1000	Abcam Cat#ab255824
Secondary antibodies	Goat anti-Rabbit IgG H&L (HRP)	1:1000	Abcam Cat#ab6721 RRID: AB_955447

### Embryoid bodies differentiation

For embryoid bodies (EB) formation, the cells were cultured in hanging drops (1,000/2,000 cells per drop) for 4 days in a DMEM (HyClone) differentiation medium containing 20% FBS (Gibco), nonessential amino acids (1 mM, Gibco), GlutaMAX (1 × , Gibco), sodium pyruvate (1 mM, Gibco), penicillin (50 units/mL), streptomycin (50 μg/mL, HyClone), and β-mercaptoethanol (0.1 mM). The EBs were then transferred into 60-mm Petri dishes and cultured in a differentiation medium for another 2–5 days. The expression of the three germ maker genes was detected on total RNA using quantitative PCR.

### Hepatocyte-like cells differentiation

The cells were cultured for 6 days on 24-well gelatin-coated plates (0.1%) at a density of 2 × 10^4^ cells/well in a differentiation medium containing 2.5 mmol/L sodium butyrate (Sigma-Aldrich, St. Louis, MO, USA), 10 ng/mL basic fibroblast growth factor (R&D Systems, Minneapolis, MN, USA), and 10 ng/mL bone morphogenetic protein 4 (R&D Systems). The medium was then replaced with a differentiation medium containing 20 ng/mL hepatocyte growth factor (R&D Systems) and incubated for another 6 days. During the last 3 days of culture, 0.1 μmol/L dexamethasone (Sigma-Aldrich) and 10 ng/mL oncostatin M (R&D Systems) was added to the medium (Fig. 3a) [17].

### RNA isolation and real-time qPCR

Total RNA was extracted using the EZ-press RNA purification kit (EZBioscience, Roseville, MN, USA), and cDNAs were synthesized using the HiScript^®^ III RT SuperMix for qPCR (Nanjing Vazyme Biotech Co., Nanjing, China). The specific gene expression was assayed using real-time qPCR (RT-qPCR) with 2× RealStar Green Power Mixture (Nanjing Vazyme Biotech Co.) on CFX Connect (Bio-Rad Laboratories, Hercules, CA, USA) with the following thermal profile: 95 °C for 10 min, followed by 40 cycles of 95 °C for 10 s, 60 °C for 10 s, and 72 °C for 20 s. The primers’ sequences used in RT-qPCR assays are listed in Table 1.

### Western blotting

The cells were lysed with radioimmunoprecipitation assay lysate and centrifuged at 12,000 RPM for 10 min at 4℃. The supernatant was collected and the protein concentration was measured. The protein sample (20 µg) was added to a 5× loading buffer, boiled at 100℃ for 10 min, and then electrophoresed in Tris–glycine buffer. The samples were then wet transferred onto a nitrocellulose membrane and blocked in 5% nonfat milk for 60 min. The membrane was washed thrice with Tris-buffered saline with Tween (TBST) for 5 min each and incubated with the primary antibody overnight at 4℃. After washing thrice with TBST for 5 min each, the membrane was incubated with the corresponding secondary antibody at room temperature for 1–2 h and washed thrice with TBST for 10 min each. Enhanced chemiluminescence solutions A and B were mixed with a volume ratio of 1:1 and dropped onto the surface of ECL in a darkroom. Exposure was made after 1 min of incubation. The antibodies used in this assay are listed in Table 2.

### Karyotyping 

The karyotyping analysis of the mutant cell line was conducted by Guangzhou Chunshui Bioscience & Technology Co. (Guangzhou, China). Twenty metaphase spreads were randomly selected for analysis, and the results revealed that the cell line generated in this study had a normal karyotype with no detectable chromosomal abnormalities.

### Cre-RNA synthesis

The Cre-RNA was synthesized using the TR101–T7 high-yield RNA transcription kit (Vazyme) following the manufacturer’s instructions and stored separately (400 ng per tube) after synthesis at − 80℃.

### Short tandem repeat analysis

The short tandem repeat (STR) analysis (Shanghai QiDa Biotechnology, Shanghai, China) revealed that the E14-F9 mutant cell line was identical to the parent cell line. The STR analysis is shown in the word document Additional file 1: “STR Analysis.”

## Results

### Vectors construction and sgRNA integration efficiency rate

The donor plasmid with the mutation was assembled according to the Golden Gate method. The screening *purinomycin* gene and red fluorescence are located between the homologous left and right arms. Loxp sequences pointing in the same direction were inserted into both ends of the screening gene (Fig. 1a), and sgRNA at different positions was selected (Fig. 1b). After plasmid construction, mouse embryonic stem cells were electrocuted and then screened in a pure medium. On the second day following the electrocution, monoclonal cells with red fluorescence were manually selected for further culture, and PCR detection was performed after amplification (Fig. 1b). The sgRNA1 sequence is as follows: CACCG TTTTTGAGTAGTACATACAC (forward) and AAAC GTGTATGTACTACTCAAAAAC (reverse). The sgRNA2 sequence is as follows: CACCGAAACTAGAAGAGTTTGTTCG (forward) and AAACCGAACAAACTCTTCTAGTTTC (reverse). The sgRNA3 sequence is as follows: CACCGAAAGGAGTGTGGAACTAGAG (forward) and AAACCTCTAGTTCCACACTCCTTTC (reverse).

sgRNA1 was off target by the detection of flanking primers. The sgRNA2 editing rate was 6.26%, with three of the 48 selected clones exhibiting the intended homologous recombination, and all three clones had mutated target sites. However, base deletion or insertion occurred at the Cas9 cleavage site because the sgRNA was located on the exon. The second Cas9 cleavage can be avoided by using the synonym mutation in the donor plasmid’s protospacer adjacent motif (PAM) region, but the PAM region of sgRNA2 only recodes glutamate. The sgRNA3 editing rate was 15.27%, with 11 of the 72 selected clones exhibiting the intended homologous recombination, which was greater than the sgRNA2 editing rate. However, given the distance between sgRNA3 and the mutation site, only one of the 11 cell clones (E14-mF9-27#) had the target-site mutation. Two bases “TA” were inserted into the intron at the site of Cas9 cutting without affecting gene expression (Fig. 1c-ii). Based on the Zhang Laboratory website [16], four off-target sites were verified, and no off-target event was detected with this study’s designed sgRNA3.

### Obtained mutant cell line

The mouse embryonic stem cells were transferred by donor and sgRNA3 and then cultured in a purinomycin environment. After passage, the surviving monoclonal cells with red fluorescence were manually selected for further PCR detection and Sanger sequencing, and the mutant cell line E14-mF9-27 # (E14-F9 C.223 C > T) was acquired (Fig. 1d). The acquired mutant was then electrotransfected with Cre-RNA to cut off the Loxp-PGK-puro-p2A-Mcherry-Loxp sequence from the genome, leaving only one Loxp sequence [18]. Following the two aforementioned gene edits, a mouse embryonic stem cell line with a human nonsense mutation, which had neither a base deletion nor an insertion in the exon but merely a single-base mutation, was obtained.

### Phenotypic analysis of mutant cell lines

**Fig. 2:**
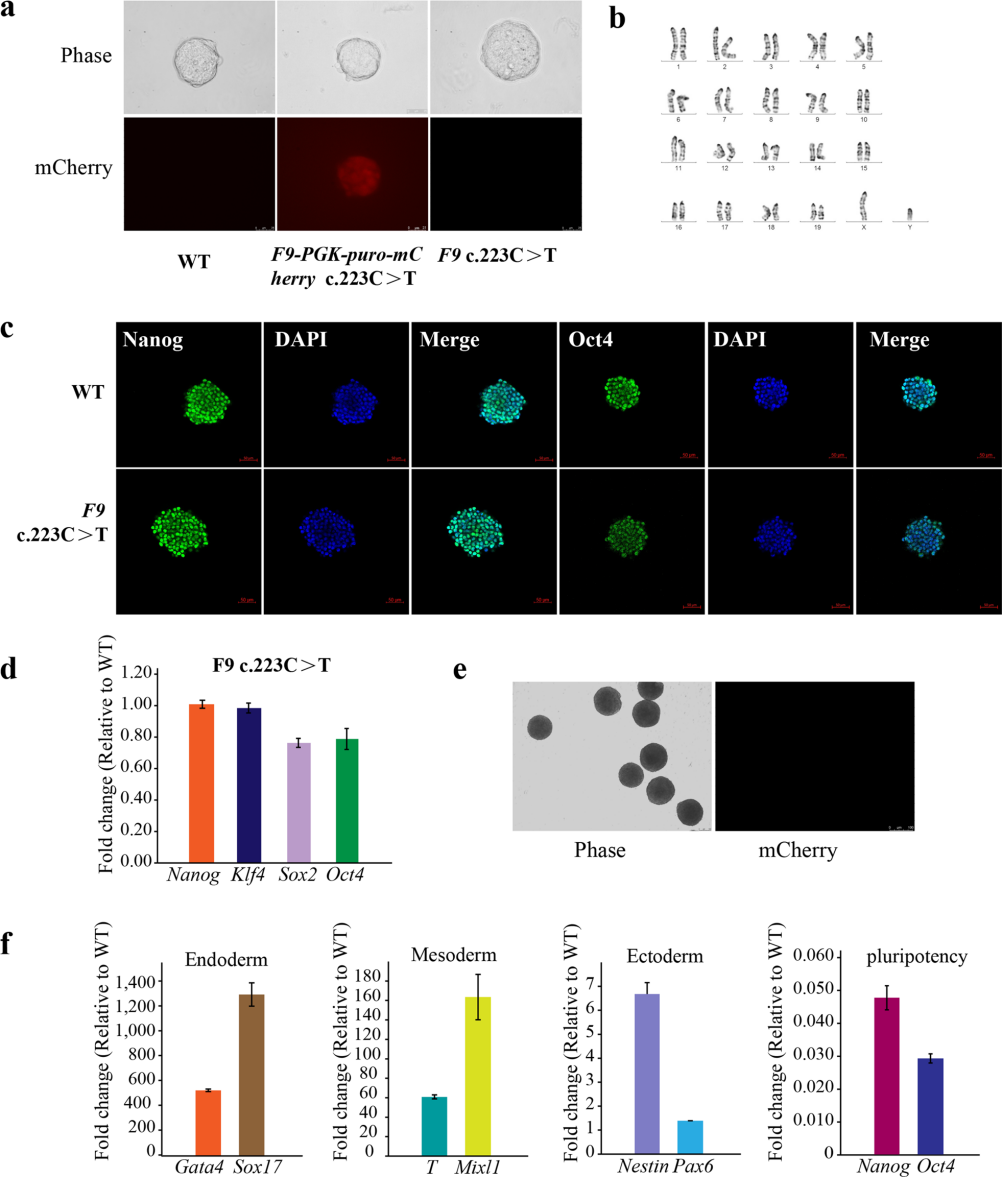
Phenotypic analysis of mutant cell lines. **a** The mutant showed a typical mESC morphology (scale, 25 μm). **b** Karyotyping was performed by G-banding, which revealed diploid 40, XY. **c** Immunofluorescence (IF) staining was used to verify the expression of multipotent markers OCT4 and NANOG (scale, 50 μm). **d** qRT-PCR was used to verify the expression of pluripotency factor RNA in the mutant and compare it to the wild type. **e**, **f** EB differentiation verifies the mutant’s ability to differentiate into three layers in vitro when compared to the wild type. **e** A morphological diagram. **f** Expression of each layer after 6 days of differentiation, as well as pluripotency loss after 6 days of differentiation

The mutant morphology is similar to that of the mouse embryonic stem cells, which is a round or nearly round monoclonal form (Fig. 2a). The karyotype analysis revealed no chromosomal abnormalities (Fig. 2b). The cell immunofluorescence staining confirmed the expression of multipotent markers NANOG and OCT4 at the protein level (Fig. 2c) and the qRT-PCR confirmed the expression of multipotent markers NANOG, Klf4, Sox2, and OCT4 at the RNA level (Fig. 2d). In addition, the differentiation ability of this mutant was verified in vitro. The EB differentiation experiments showed that the EBs were developed (Fig. 2e) and the markers of all three dermal layers were highly expressed. Furthermore, pluripotency was lost after EB differentiation (Fig. 2f). In conclusion, the mutant that was engineered in the current study retained the characteristics of mouse embryonic stem cells, pluripotency, and three layers of differentiation potential.

### The effect of point mutation on *F9* gene function in differentiation system

**Fig. 3:**
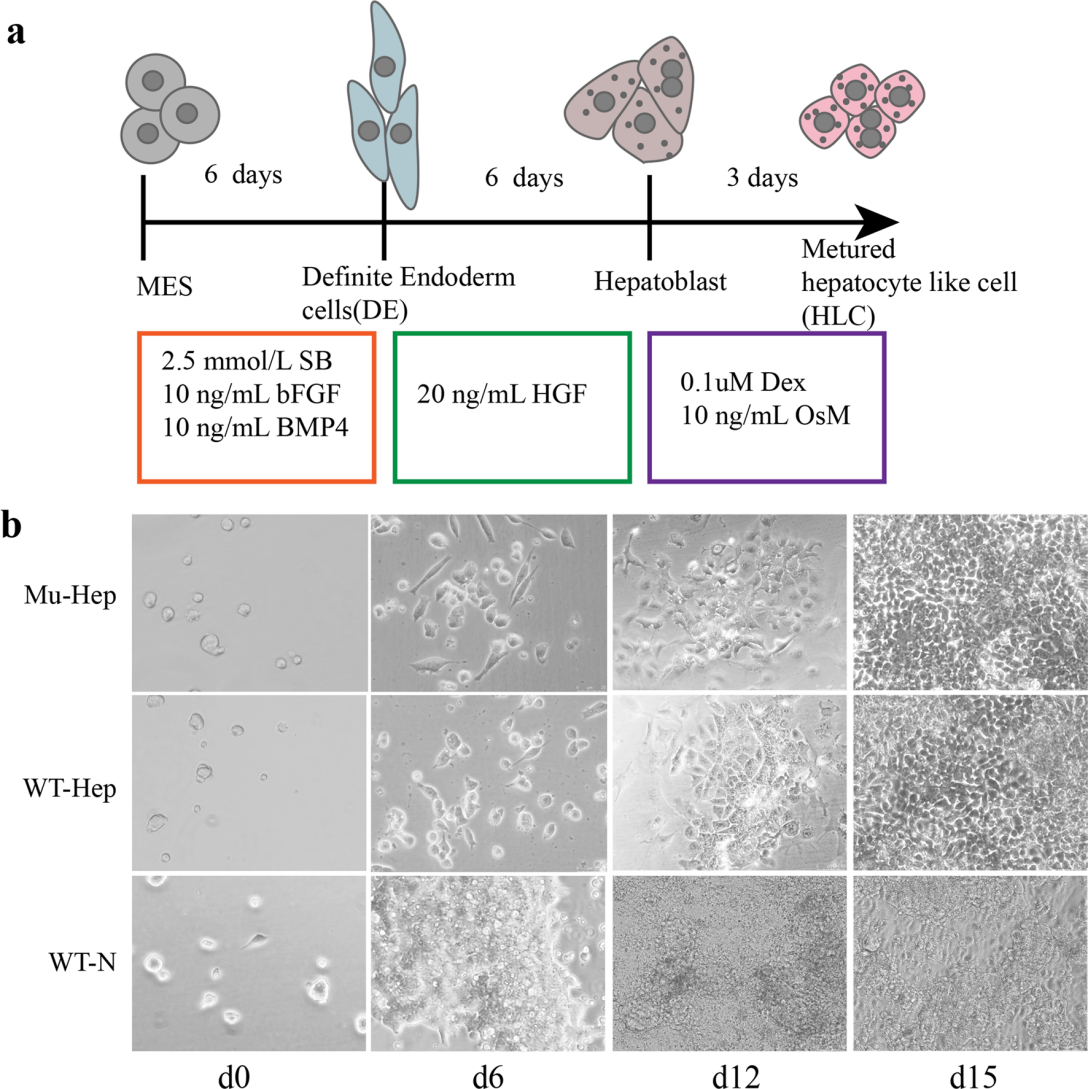
Differentiation of hepatocyte-like cells. **a** A diagram of differentiation patterns. **b** Morphological changes of cells during differentiation

**Fig. 4:**
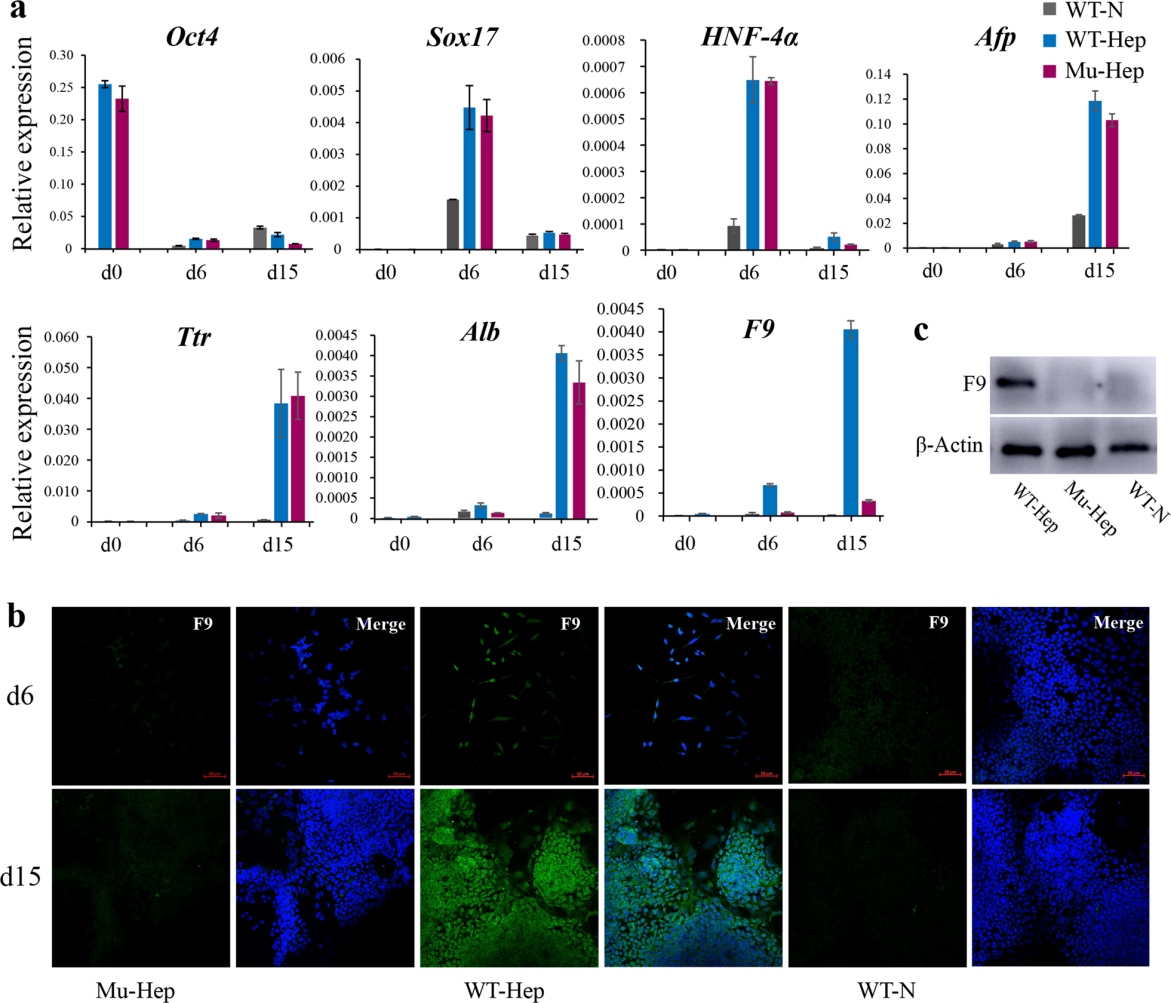
Marker detection in hepatoid cells. **a** RNA expression levels of various markers at days 0, 6, and 15 of differentiation, **b** immunofluorescence staining after the 15th day of differentiation, and **c** protein expression on the 15th day of differentiation

The effect of this point mutation on gene function in the differentiation system was investigated. The *F9* gene was found to be mostly expressed in the mature liver with minimal expression in the fetal liver and no expression in embryonic stem cells [19]. Previous studies have demonstrated that induced hepatocyte-like cells can express coagulation factors VIII and IX [17, 20]. Embryonic stem cells were differentiated into hepatocyte-like cells following the previously described protocol [17] (Fig. 3a) and observed cell morphological changes during differentiation. The cells change from a round stem cell shape to a fusiform-defined endoderm shape and eventually differentiate into polygonal hepatoid cells. No obvious morphological difference was noted between mutant (Mu-Hep) and nonmutant (WT-hep) groups, but the cell morphology of the naturally differentiated group (WT-N) was different and irregular (Fig. 3b). On day 15 of differentiation, albumin (ALB), a specific markers of hepatocyte-like cells, could be detected in Mu-Hep and WT-Hep, but *F9* expression was detected only in WT-Hep (Fig. 4a–c). In addition, point mutations do not affect hepatocyte-like cell differentiation but affect *F9* gene transcription and translation, similar to the human patient with this mutation, but the patient was a severe hemophiliac.

## Discussion

The majority of models used to study hemophilia in research are hemophilia gene deletion models. Gene therapy aims to achieve the treatment goals by inserting normal foreign genes into the cell genome to replace defective ones. The insertion of foreign genes into the genome necessitates the use of vectors, which trigger an immunological reaction in the host. This is a significant barrier in gene therapy; thus, endogenous vectors should be explored [21]. In addition, CRISPR gene editing can introduce hundreds of unpredictable mutations into the genome, posing serious hurdles for clinical use [22, 23]. Many types of hemophilia gene mutations exist, and the gene deletion model cannot adapt to the specific mutation mechanism. In the present study, a nonsense mutation model that does not affect cell proliferation or development but only affects *F9* gene function, which is comparable to clinical instances, was developed. This model is appropriate for studying the hemophilia nonsense mutation mechanism and personalized medicine.

The current study found that when the sgRNA is close to the mutation site, it favors mutation. This condition is related to the DNA repair mechanism, generating double-bond breaks on sgRNA, resulting in gene repair and dramatically increasing mutations [24]. In addition, the efficiency of gene editing integration is related to the chromosome/chromatin state and sgRNA activity [25, 26].

In EB differentiation, the expression of endoderm markers is elevated, which is consistent with previous studies [27, 28]. This mechanism is consistent with the process of embryonic development. In the early stages of embryonic development, the endoderm and mesoderm develop first, followed by the ectoderm. The expression of endodermal and mesodermal markers was high as detected on the sixth day of EB differentiation, which is related to early embryonic development, while the expression of neuroectodermal markers was low because nerve development is known to be relatively late [29, 30]. This result also demonstrates that the mutant cell line has the normal potential to differentiate into three layers in vitro.

In the hepatocyte-like cell differentiation system, efficiency was not evident although the current study differentiated mESCs into hepatocyte-like cells, and the expression level of liver markers was lower than that previously reported [31, 32]. On the one hand, the presence of undifferentiated cells in the sample was considered without segregating them from differentiated cells. However, the maturity of differentiated hepatocytes in vitro is not high, and the expression level of markers is close to that in the fetal liver, which is quite different from the mature liver [33]. Overall, point mutations were indicated to affect gene function, comparable to the human patient with this mutation, who is a severe hemophiliac [16]. However, the mechanism of the disease remains unknown, and, therefore, future studies are warranted.

## Conclusions

The mutant cell line obtained in the present study did not have any base deletion or insertion in the exon but merely a single-base mutation, which is closer to clinical cases. The growth capacity and cell morphology of the mutant were comparable to those of the wild type. The immunofluorescence staining and RT-qPCR findings revealed that the mutant could express pluripotency markers at both the protein and RNA levels. In vitro free differentiation showed that the mutant could differentiate into three layers. After hepatocyte-like cell differentiation, the gene mutation, like the human gene mutation, was confirmed to affect the *F9* gene function. In conclusion, the mutant has the characteristics of mouse embryonic stem cells and can be used as a disease model for studying the pathogenesis and treatment of hemophilia at the cellular level. It can also be used for chimeric injection of mouse embryonic stem cells to obtain a mouse model of hemophilia, providing the groundwork for future studies into the disease.

## Supplementary Information

**Additional file 1.** STR Analysis.

**Additional file 2.** Text 1, and the primers’ sequencesare listed in Table 1.

## Data Availability

All data will be made available upon reasonable request to the corresponding authors.

## Abbreviations

PAM: Protospacer adjacent motifs
EB: Embryoid body
CRISPR: Clustered regularly interspaced short palindromic repeats
FIX: Clotting factor IX
PTCs: Premature termination codons
mESC: Mouse embryonic stem cell

## References

[1] Ghaemi A, Bagheri E, Abnous K, Taghdisi SM, Ramezani M, Alibolandi M (2021). CRISPR-cas9 genome editing delivery systems for targeted cancer therapy. Life Sci.

[2] Mali P, Esvelt KM, Church GM (2013). Cas9 as a versatile tool for engineering biology. Nat Methods.

[3] Berntorp E, Fischer K, Hart DP, Mancuso ME, Stephensen D, Shapiro AD (2021). Haemophilia. Nat Rev Dis Primers.

[4] Berntorp E, Shapiro AD (2012). Modern haemophilia care. Lancet.

[5] Biggs R, Macfarlane RG (1958). Haemophilia and related conditions: a survey of 187 cases. Br J Haematol.

[6] White GC, Rosendaal F, Aledort LM, Lusher JM, Rothschild C, Ingerslev J (2001). Definitions in haemophilia. Recommendation of the scientific subcommittee on factor VIII and factor IX of the scientific and standardization committee of the International Society on Thrombosis and Haemostasis. Thromb Haemost.

[7] Srivastava A, Santagostino E, Dougall A, Kitchen S, Sutherland M, Pipe SW (2020). WFH guidelines for the management of hemophilia, 3rd edition. Haemophilia.

[8] Kempton CL, Meeks SL (2014). Toward optimal therapy for inhibitors in hemophilia. Blood.

[9] Gouw SC, van den Berg HM, Oldenburg J, Astermark J, de Groot PG, Margaglione M (2012). F8 gene mutation type and inhibitor development in patients with severe hemophilia A: systematic review and meta-analysis. Blood.

[10] Mort M, Ivanov D, Cooper DN, Chuzhanova NA (2008). A meta-analysis of nonsense mutations causing human genetic disease. Hum Mutat.

[11] Brogna S, Wen J (2009). Nonsense-mediated mRNA decay (NMD) mechanisms. Nat Struct Mol Biol.

[12] Keeling KM, Bedwell DM (2011). Suppression of nonsense mutations as a therapeutic approach to treat genetic diseases. Wiley Interdiscip Rev RNA.

[13] Hogg JR, Goff SP (2010). Upf1 senses 3'UTR length to potentiate mRNA decay. Cell.

[14] Linde L, Boelz S, Nissim-Rafinia M, Oren YS, Wilschanski M, Yaacov Y (2007). Nonsense-mediated mRNA decay affects nonsense transcript levels and governs response of cystic fibrosis patients to gentamicin. J Clin Investig.

[15] Keeling KM, Xue X, Gunn G, Bedwell DM (2014). Therapeutics based on stop codon readthrough. Annu Rev Genomics Hum Genet.

[16] http://www.factorix.org/advance_search_results.php.

[17] Cao J, Shang CZ, Lü LH, Qiu DC, Ren M, Chen YJ (2010). Differentiation of embryonic stem cells into hepatocytes that coexpress coagulation factors VIII and IX. Acta Pharmacol Sin.

[18] Yang F, Liu C, Chen D, Tu M, Xie H, Sun H (2017). CRISPR/Cas9-loxP-mediated gene editing as a novel site-specific genetic manipulation tool. Mol Ther Nucleic Acids.

[19] Baxter M, Withey S, Harrison S, Segeritz CP, Zhang F, Atkinson-Dell R (2015). Phenotypic and functional analyses show stem cell-derived hepatocyte-like cells better mimic fetal rather than adult hepatocytes. J Hepatol.

[20] Meng Y, Huang S, Min J, Guo Z (2006). In vitro differentiation of mouse ES cells into hepatocytes with coagulation factors VIII and IX expression profiles. Sci China Ser C Life Sci.

[21] Segel M, Lash B, Song J, Ladha A, Liu CC, Jin X (2021). Mammalian retrovirus-like protein PEG10 packages its own mRNA and can be pseudotyped for mRNA delivery. Science.

[22] Schaefer KA, Wu WH, Colgan DF, Tsang SH, Bassuk AG, Mahajan VB (2017). Unexpected mutations after CRISPR-Cas9 editing in vivo. Nat Methods.

[23] Smith RH, Chen YC, Seifuddin F, Hupalo D, Alba C, Reger R (2020). Genome-wide analysis of off-target CRISPR/Cas9 activity in single-cell-derived human hematopoietic stem and progenitor cell clones. Genes.

[24] Chatterjee N, Walker GC (2017). Mechanisms of DNA damage, repair, and mutagenesis. Environ Mol Mutagen.

[25] Hiranniramol K, Chen Y, Wang X (2020). CRISPR/Cas9 guide RNA design rules for predicting activity. Methods Mol Biol.

[26] Doench JG, Fusi N, Sullender M, Hegde M, Vaimberg EW, Donovan KF (2016). Optimized sgRNA design to maximize activity and minimize off-target effects of CRISPR-Cas9. Nat Biotechnol.

[27] Ma Y, Sun W, Liu X, Ren J, Zhang X, Zhang R (2022). Generation an induced pluripotent stem cell line SXMUi001-A derived from a hemophilia B patient carries variant F9 c.223C>T(p.R75X). Stem Cell Res.

[28] Yao M, Su P, Li Z, Cui X, Yang Q, Xing X (2021). Knockout of Dip2c in murine ES cell line IBMSe001-B-1 by CRISPR/Cas9 genome editing technology. Stem Cell Res.

[29] Barzegari A, Gueguen V, Omidi Y, Ostadrahimi A, Nouri M, Pavon-Djavid G (2020). The role of Hippo signaling pathway and mechanotransduction in tuning embryoid body formation and differentiation. J Cell Physiol.

[30] Zeevaert K, Elsafi Mabrouk MH, Wagner W, Goetzke R (2020). Cell mechanics in embryoid bodies. Cells.

[31] Luce E, Steichen C, Allouche M, Messina A, Heslan JM, Lambert T (2022). In vitro recovery of FIX clotting activity as a marker of highly functional hepatocytes in a hemophilia B iPSC model. Hepatology (Baltimore, MD).

[32] Wang Q, Sun D, Liang Z, Wang J, Zhong X, Lyu Y (2020). Generation of human hepatocytes from extended pluripotent stem cells. Cell Res.

[33] Li Y, Yang X, Plummer R, Hayashi Y, Deng XS, Nie YZ (2021). Human pluripotent stem cell-derived hepatocyte-like cells and organoids for liver disease and therapy. Int J Mol Sci.

