# Supplementary material for: Generation of an mESC model with a human hemophilia B nonsense mutation via CRISPR/Cas9 technology
Source: Stem Cell Res Ther. 2022 Jul 26;13:353. doi: 10.1186/s13287-022-03036-2 (PMC9327398; doi:10.1186/s13287-022-03036-2)

| Loci | STR information of simple cells | | STR information of cell bank | |
| --- | --- | --- | --- | --- |
|  | E14-F9 c.223C＞T | | E14 | |
|  | Allele1 | Allele2 | Allele1 | Allele2 |
| 4-2 | 229.46 【18.3】 |  | 229.65 |  |
| 5-5 | 335.49  【14】 |  | 335.79 |  |
| 6-4 | 299.82  【18】 |  | 299.96 |  |
| 6-7 | 334.14  【12】 |  | 334.75 |  |
| 9-2 | 225.05  【16】 |  | 225.32 |  |
| 12-1 | 229.59  【17】 |  | 229.83 |  |
| 15-3 | 192.62 【20.3】 |  | 192.83 | 229.83 |
| 18-3 | 155.76  【17】 | 159.86  【18】 | 155.77 | 159.81 |
| X-1 | 404.17  【26】 |  | 404.4 |  |

STR analysis of E14-F9 c.223C＞T


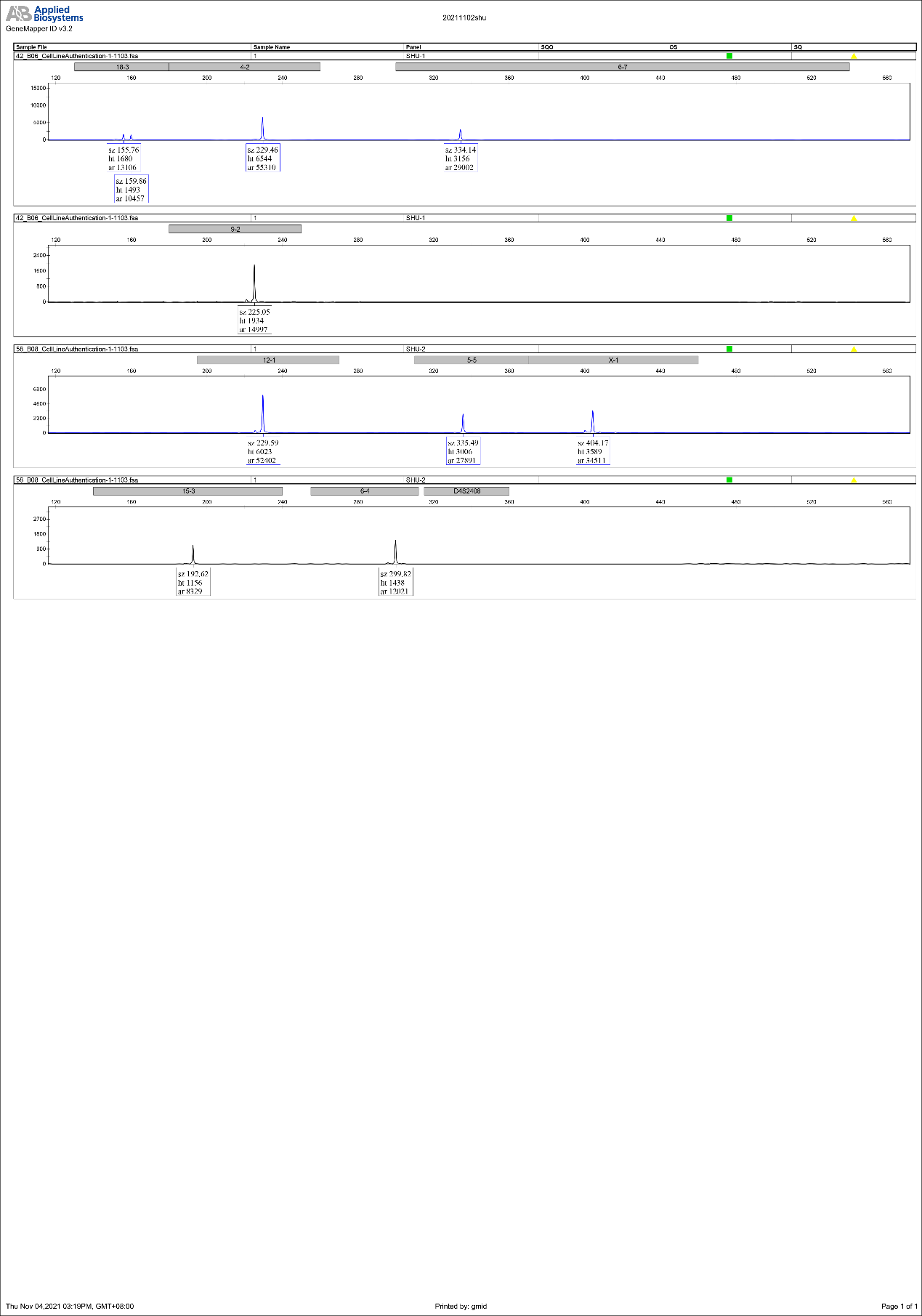

Supplement: Supplementary file 1 — Additional file 1. STR Analysis. [file 13287_2022_3036_MOESM1_ESM.docx]
