# Supplementary material for: Generation of an mESC model with a human hemophilia B nonsense mutation via CRISPR/Cas9 technology
Source: Stem Cell Res Ther. 2022 Jul 26;13:353. doi: 10.1186/s13287-022-03036-2 (PMC9327398; doi:10.1186/s13287-022-03036-2)
Supplement: Supplementary file 2 — Additional file 2. Text 1, and the primers’ sequencesare listed in Table 1. [file 13287_2022_3036_MOESM2_ESM.docx]

Supplementary Text1.

LA: caaacaggcttctgtccttcgcgggatatttgttgcaaagtcattgggagactacacactggaaacagcccagccagaggaatgatagatcaccttggaacgatcctgtactgagctattttcaagagatgacaaagtgggaacttaactgcttagttgagaactttctttttcatccaaagttaaacacaaatacaactgaaatgtaaccttttattgctggcactctaccaattaaaaataaaattgaattttaattcctaaatttccatgtgtatactgtgaaaaaaaaatcatagtttttggctctatgccccaaagagaaattagctatggaattacatgaacccaaaacaaaccttttctttaaaaacatatttagttttctgaggtttttttttttcctaatactaaagaactatacttttaaatttcagttttccttgatcgtgaaaatgccaccaaaattcttacccgtccaaagagatataattcaggaaaactagaagagtttgttcgaggaaaccttgaaagagagtgtatagaagaaagatgtagttttgaagaagcatgagaagtttttgaaaacactgaaaaaactgtgagtatacccacatcatacctgaataatatgtcccagagcaaaagatagaaaatctctattttcaaaaggagtgtggaactagagaggtgactccatatttaggagcactctgctattccagagcacacagattcaaattcaagcatctaggaaacccaacactctcttctgacctttttgagtagtacatacacagg

Loxp-PGK-puro-P2A-mCherry-Loxp:

ataacttcgtataatgtatgctatacgaagttatgggtaggggaggcgcttttcccaaggcagtctggagcatgcgctttagcagccccgctgggcacttggcgctacacaagtggcctctggcctcgcacacattccacatcccccggtaggcgccaaccggctccgttctttggtggccccttcgcgccaccttctactcctcccctagtcaggaagttcccccccgccccgcagctcgcgtcgtgcaggacgtgacaaatggaagtagcacgtctcactagtctcgtgcagatggacagcaccgctgagcaatggaagcgggtaggcctttggggcagcggccaatagcagctttgctccttcgctttctgggctcagaggctgggaaggggtgggtccgggggcgggctcaggggcgggctcaggggcggggcgggcgcccgaaggtcctccggaggcccggcattctgcacgcttcaaaagcgcacgtctgccgcgctgttctcctcttcctcatctccgggcctttcgacctgcagcccaagctagcttaccatgaccgagtacaagcccacggtgcgcctcgccacccgcgacgacgtccccagggccgtacgcaccctcgccgccgcgttcgccgactaccccgccacgcgccacaccgtcgatccggaccgccacatcgagcgggtcaccgagctgcaagaactcttcctcacgcgcgtcgggctcgacatcggcaaggtgtgggtcgcggacgacggcgccgcggtggcggtctggaccacgccggagagcgtcgaagcgggggcggtgttcgccgagatcggcccgcgcatggccgagttgagcggttcccggctggccgcgcagcaacagatggaaggcctcctggcgccgcaccggcccaaggagcccgcgtggttcctggccaccgtcggcgtctcgcccgaccaccagggcaagggtctgggcagcgccgtcgtgctccccggagtggaggcggccgagcgcgccggggtgcccgccttcctggagacctccgcgccccgcaacctccccttctacgagcggctcggcttcaccgtcaccgccgacgtcgaggtgcccgaaggaccgcgcacctggtgcatgacccgcaagcccggtgccgccactaacttcagcttgttgaagcaggccggagacgtcgaagagaacccgggtccaatggtgagcaagggcgaggaggataacatggccatcatcaaggagttcatgcgcttcaaggtgcacatggagggctccgtgaacggccacgagttcgagatcgagggcgagggcgagggccgcccctacgagggcacccagaccgccaagctgaaggtgaccaagggtggccccctgcccttcgcctgggacatcctgtcccctcagttcatgtacggctccaaggcctacgtgaagcaccccgccgacatccccgactacttgaagctgtccttccccgagggcttcaagtgggagcgcgtgatgaacttcgaggacggcggcgtggtgaccgtgacccaggactcctccctgcaggacggcgagttcatctacaaggtgaagctgcgcggcaccaacttcccctccgacggccccgtaatgcagaagaagacgatgggctgggaggcctcctccgagcggatgtaccccgaggacggcgccctgaagggcgagatcaagcagaggctgaagctgaaggacggcggccactacgacgctgaggtcaagaccacctacaaggccaagaagcccgtgcagctgcccggcgcctacaacgtcaacatcaagttggacatcacctcccacaacgaggactacaccatcgtggaacagtacgaacgcgccgagggccgccactccaccggcggcatggacgagctgtacaagtaaataacttcgtataatgtatgctatacgaagttat

RA:

Gtgcatgtatgcacacagatagaacactcatacacataaaacaaaatacaaagtattatagatcaattattaaaggaaaattgtatttcaaatcttaaaaatatcagttcatatcatatcatctattacaaatgttctaaaaggaaaggaatccatatcaaaatactttaaacactatcattaagctgtcctcctttttccttacagactgaattttggaagcagtatgttggtaagcaattcattttattttattttatttcctacctgctatatgaaacacttgagaattgtgccttttttctatatagagaggttgtacagtctcagtaaaaaaaaaaaaaatcaggaaaaaaaccaaacaactgcatcttagagctaaatgtacatttactgtaactagtagattcagagatgattgggatggtttccaatgccctccgtgtcttgacctaccatcccttcttgttgccctctgaccacctctactactactgccctcattccacaaaggggctccttgcatgccctagcactgttcccatattagaggaacaatcttactatccctgttagctgagtcttcccagcttcttctaagctaacttccttaccttgctcagggacttgtccaaaattcctccaactcagtaagtcctcccttgataaggtattaatattccaacctgctacctggtcgctctgtcattttagacacatgtgttctggtc
